# Epidermal Transglutaminase (TGase 3) Is Required for Proper Hair Development, but Not the Formation of the Epidermal Barrier

**DOI:** 10.1371/journal.pone.0034252

**Published:** 2012-04-04

**Authors:** Susan John, Lars Thiebach, Christian Frie, Sharada Mokkapati, Manuela Bechtel, Roswitha Nischt, Sally Rosser-Davies, Mats Paulsson, Neil Smyth

**Affiliations:** 1 Center for Biochemistry, University of Cologne, Cologne, North Rhine-Westphalia, Germany; 2 Center for Molecular Medicine (CMMC), University of Cologne, Cologne, North Rhine-Westphalia, Germany; 3 Department of Dermatology, University of Cologne, Cologne, North Rhine-Westphalia, Germany; 4 The School of Biological Sciences, University of Southampton, Southampton, United Kingdom; 5 Cologne Excellence Cluster on Cellular Stress Responses in Aging-associated Diseases (CECAD), Cologne, North Rhine-Westphalia, Germany; University Hospital Hamburg-Eppendorf, Germany

## Abstract

Transglutaminases (TGase), a family of cross-linking enzymes present in most cell types, are important in events as diverse as cell-signaling and matrix stabilization. Transglutaminase 1 is crucial in developing the epidermal barrier, however the skin also contains other family members, in particular TGase 3. This isoform is highly expressed in the cornified layer, where it is believed to stabilize the epidermis and its reduction is implicated in psoriasis. To understand the importance of TGase 3 *in vivo* we have generated and analyzed mice lacking this protein. Surprisingly, these animals display no obvious defect in skin development, no overt changes in barrier function or ability to heal wounds. In contrast, hair lacking TGase 3 is thinner, has major alterations in the cuticle cells and hair protein cross-linking is markedly decreased. Apparently, while TGase 3 is of unique functional importance in hair, in the epidermis loss of TGase 3 can be compensated for by other family members.

## Introduction

Protein cross-links are crucial for many developmental and regenerative events. Extracellularly they stabilize the extracellular matrix, while intracellular cross-linking contributes to the formation and strengthening of epithelial barriers. Transglutaminases catalyze the formation of Nε-(γ-glutamyl)lysine isopeptide bonds between amino acid side-chains [1]. This Ca^2+^-dependent reaction results in formation of a bond between a glutamine side chain and an amine donor. Where the latter is a lysine residue within a protein, this results in inter- or intramolecular cross-links and may form stable supramolecular structures [2]. The glutamine residues used as substrates are restricted [3], [4], with specificity presumably depending on surrounding conformation and charge [5]. Thus each transglutaminase has a range of substrates that varies between the individual enzymes [6]. Although transglutaminases lack a conventional secretory signal peptide they are released and are active extracellularly [7]. Here they stabilize the surrounding matrix or in the case of the blood coagulation factor XIIIa, the fibrin clot. Of the transglutaminases present in different compartments of the skin, three have been studied in greater detail. TGase 1 (keratinocyte transglutaminase), TGase 3 (epidermal transglutaminase) and TGase 5 have all been suggested to play important roles in epidermal keratinization and in the formation of the cornified envelope [8]–[10]. TGase 2 (tissue transglutaminase) is believed to aid dermal matrix remodelling during wound healing [11]. Natural mutations and gene targeting studies indicate that certain transglutaminases play unique roles which cannot or can only be partially compensated for in their absence. Thus, alterations in membrane bound TGase 1 cause the autosomal recessive skin disorder *lamellar ichthyosis* [12], [13]. This occurs despite the expression of other transglutaminases in differentiating keratinocytes. Patients with *lamellar ichthyosis* have an abnormal *stratum corneum*, with its cells retaining organelles normally lost during keratinization. Further the lipid and cornified envelopes do not form, leading to a severely compromised skin barrier [14]. TGase 3 is a soluble enzyme expressed predominantly in differentiating keratinocytes, corneocytes and hair follicles [15], and it is also found extracellularly [16]. Biochemical assays with skin extracts suggest that TGase 3 is responsible for much of the transglutaminase activity in the epidermis [17]. The full range of possible proteases activating this zymogen *in vivo* still needs further evaluation but a major candidate for an *in vivo* role is cathepsin L [18]. However, a number of enzymes cause *in vitro* activation. Structural studies show that cleavage does not result in the dissociation of the subunits, rather it allows Mg^2+^ binding. Upon increase of intracellular Ca^2+^ levels, for instance during epidermal differentiation, Ca^2+^ displaces Mg^2+^ resulting in the induction of a conformational change. This leads to unmasking of the active site allowing interaction with the first substrate [19]. Upon substrate interaction large conformational changes occur [20]. There are many transglutaminase substrates in differentiating keratinocytes, including loricrin and involucrin [8]. These are cross-linked to different degrees by the various transglutaminases [9]. For instance, while TGase 1 induces loricrin multimerization at the inner surface of the plasma membrane, TGase 3 causes intramolecular linkage or dimerization of loricrin in the cytoplasm [8]. Assembly of the cornified envelope is a highly ordered event, though surprisingly, loss of certain major structural proteins including involucrin and loricrin results in remarkably little change in its formation [21], [22]. The differential localization of the epidermal transglutaminases, both with respect to the stages of keratinocyte differentiation and to their intracellular localization, combined with their different substrate usage, strongly suggests highly specific roles for these enzymes in keratinization. Trichohyalin is a glutamine-rich protein highly expressed in specialized epithelial structures with high mechanical strength [23], such as the inner root sheath which forms a collar about the hair. It is a substrate for TGase 3 and the *in vivo* expression of TGase 3 coincides with the modification of trichohyalin [24]. This finding is in agreement with *in vitro* results showing that TGase 1 or TGase 2, which are also present in the inner root sheath, cross-link trichohyalin to a far lesser degree [24], [25]. The transglutaminase-modified trichohyalin is believed to serve as a matrix onto which keratin intermediate filaments are deposited. To understand the importance of TGase 3 in development and maintenance of tissues, we produced and analyzed the phenotype of a novel mouse line lacking this enzyme.

## Results

### Generation of mice lacking TGase 3

**Figure 1:**
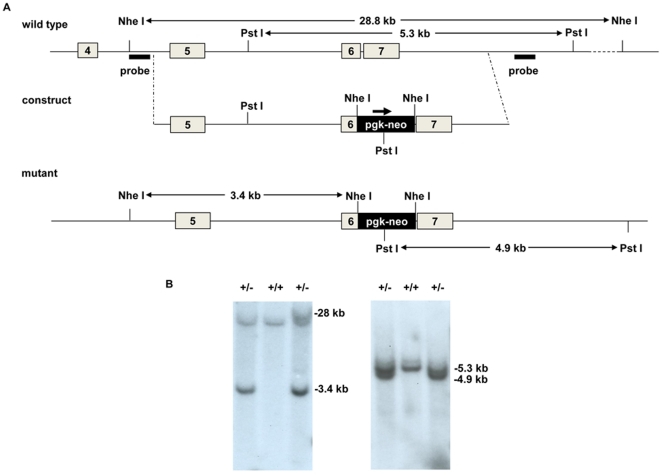
Ablation of the *TGM3* gene. The locus of exons 4 to 7 of the *TGM3* gene (A). The targeting construct was produced by insertion of the neomycin resistance cassette into exon 6. Southern blot analysis of cells after restriction digestion using NheI for the 5′ probe and PstI with the 3′ probe was used to identify correctly targeted ES cells. The disruption of exon 6 resulted in the wild type 28.8 kb NheI fragment being reduced to 3.4 kb, and the 5.3 kb PstI fragment being reduced to 4.9 kb. Southern blot analysis of ES cell DNA after NheI digestion and hybridization with the 5′ probe (B, left panel), and after PstI digestion and hybridization with the 3′ probe (B, right panel).

**Figure 2:**
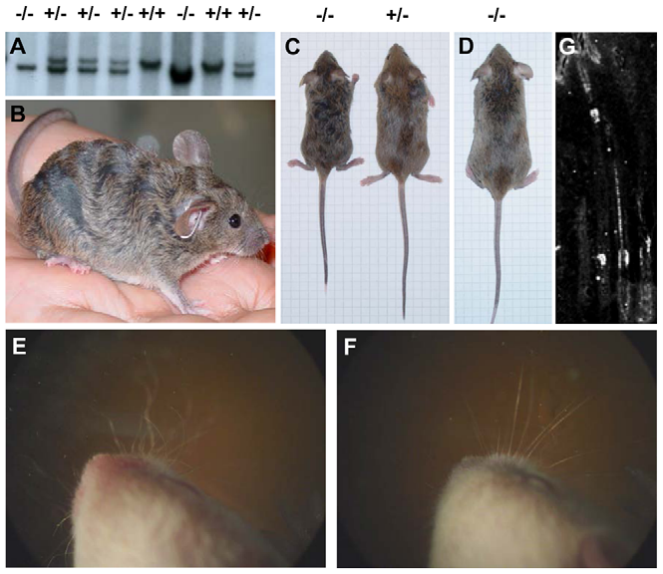
Gross phenotype of *TGM3* null mice. Southern blot analysis of tail biopsies from mice born of *TGM3^+/−^* interbreeding showed that homozygous mutants were born at the expected Mendelian ratios (A). The pelage hair of homozygous animals at 4 weeks of age showed a distinctive wavy pattern (B and C). This became less obvious as the mice matured (D). While gross hair abnormalities disappear with time, irregularities in the vibrissae, which are evident perinatally (E, mutant; F, control at P5) persist throughout life. Expression of TGase 3 is observed in pelage hair in the medulla and in the inner root sheath (G).

**Figure 3:**
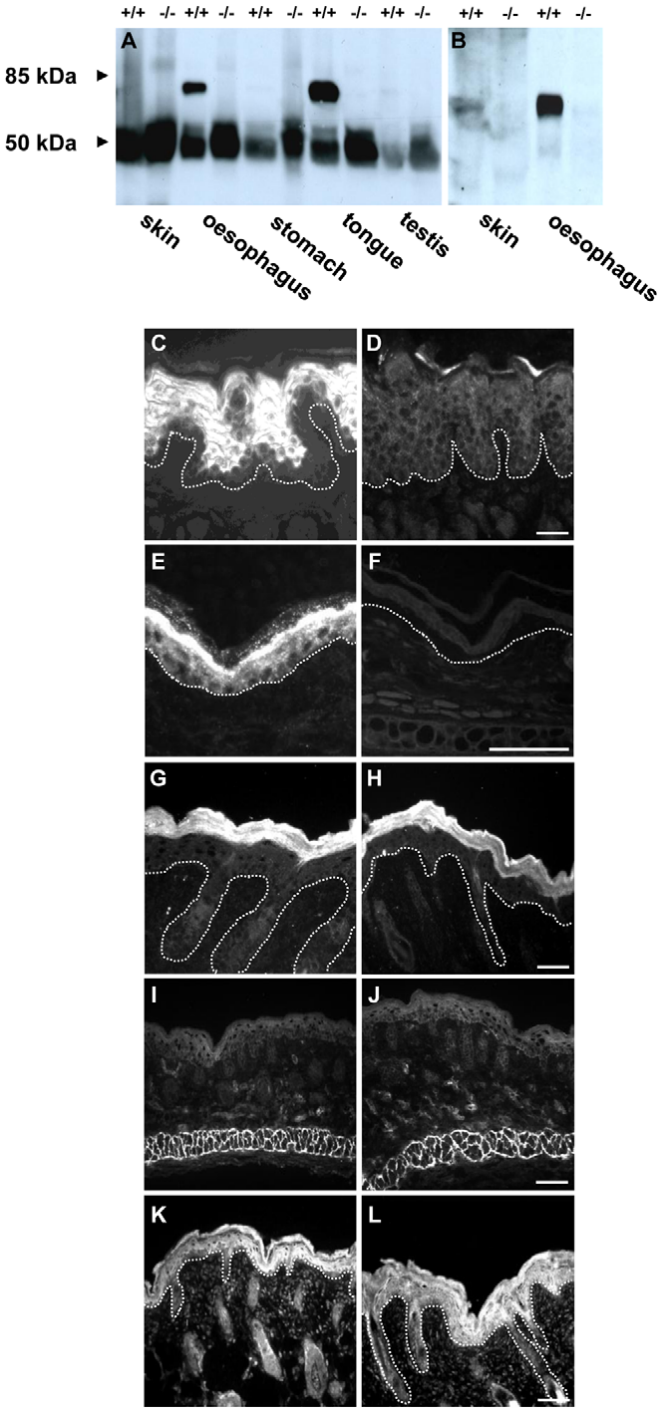
TGase 3 expression in epithelia. Upper panel, protein extracts separated by SDS-PAGE were incubated with a mouse monoclonal antibody (A) and a rabbit polyclonal antiserum (B) against TGase 3. The expected TGase 3 band of 77 kDa was observed in extracts from wild type animals and was especially strong in oesophagus and tongue epithelium. In protein extracts from homozygous animals the signal was completely absent. The 50 kDa band seen in (A) corresponds to the heavy chain of IgG. Lower panel, immunofluorescence analysis of tongue (C, D) and back skin (E–L) of wild type (C, E, G, I, K) and *TGM3* ^−/−^ animals (D, F, H, J, L). The sections were incubated with rabbit polyclonal antibodies against TGase 3 (C–F), TGase 6 (K and L) and monoclonal antibodies against TGase 1 (G, H) and TGase 2 (I and J). (scale bars represent 100 µm, the dotted line marks the dermal-epidermal junction).

Embryonic stem cells were targeted at the *TGM3* allele by disrupting the sixth exon and introducing a neomycin resistance gene (Fig. 1A). This destroys the active center of the enzyme and splicing of exon 5 to any of the following exons (7 to 11) will result in an out of frame product and/or an unstable mRNA transcript. Three clones were correctly targeted when analyzed by Southern blotting and probing 5′, 3′ and internal to the targeting site (Fig. 1B). Two of these were used to derive chimeras by injection of cells into C57/Bl6 blastocysts, and germ-line transmission was obtained. Breeding mice heterozygous for the *TGM3* mutation produced offspring with the three possible genotypes at the expected Mendelian ratios (Fig. 2A), showing that mutation of the *TGM3* allele is compatible with life. Western blotting and probing either with a mouse monoclonal antibody or with an affinity purified antiserum against recombinant TGase 3 verified the absence of the protein, with the expected 77 kDa band being absent in mutant tissues (Fig. 3A and B). Further, RT-PCR with *TGM3* specific primers annealing 5′ and 3′ to exon 6 failed to amplify any product (Fig. S1), suggesting destabilization of the TGase 3 mRNA and a null mutation in the *TGM3* gene. *TGM3* ^−/−^ males and females display defects in hair development but are fertile and have a normal lifespan. (Fig. 2).

### *TGM3^−/−^* mice do not display changes in epidermal differentiation and develop a normal cornified envelope

Since TGase 3 has been described as a major contributor of transglutaminase activity in the epidermis [23], which is a crucial factor in the development and stabilization of the skin, we initially carried out an extensive analysis of the epidermis. Grossly, the skin of null mice appeared normal but there was an obvious alteration in hair development (see below). Histology showed no major changes (Fig. S2) nor was there evidence for increased scaling. The apparent lack of changes led us to suppose that there could be a compensation for the loss of TGase 3. However, immunostaining of skin with antibodies raised against TGase 1, TGase 2 or TGase 6, the closest relative to TGase 3, showed no alteration in the expression pattern of these main skin transglutaminases (Fig. 3C–L). Interestingly, TGase 2 which is present in human basal keratinocytes, was not detected in mouse epidermis, although it was seen staining the epimysium (Fig. 3I and J). Expression of TGase 5 at the mRNA level was also unaltered (Fig. S3). TGase 3 is present in keratinizing compound squamous epithelia, yet there were no gross abnormalities of the oesophagus, filiform papillae of the tongue or the keratinized stomach and histology also failed to reveal any changes (Fig. S2). We then studied expression of markers for keratinocyte differentiation. These included keratin isoforms, filaggrin, loricrin and involucrin, all known substrates for TGase 3 and the latter two being highly expressed in the *stratum granulosum*. None showed an altered expression pattern (Fig. S2).

### Absence of TGase 3 does not affect epidermal barrier function in newborn or adult mice despite changes in corneocyte stability

**Figure 4:**
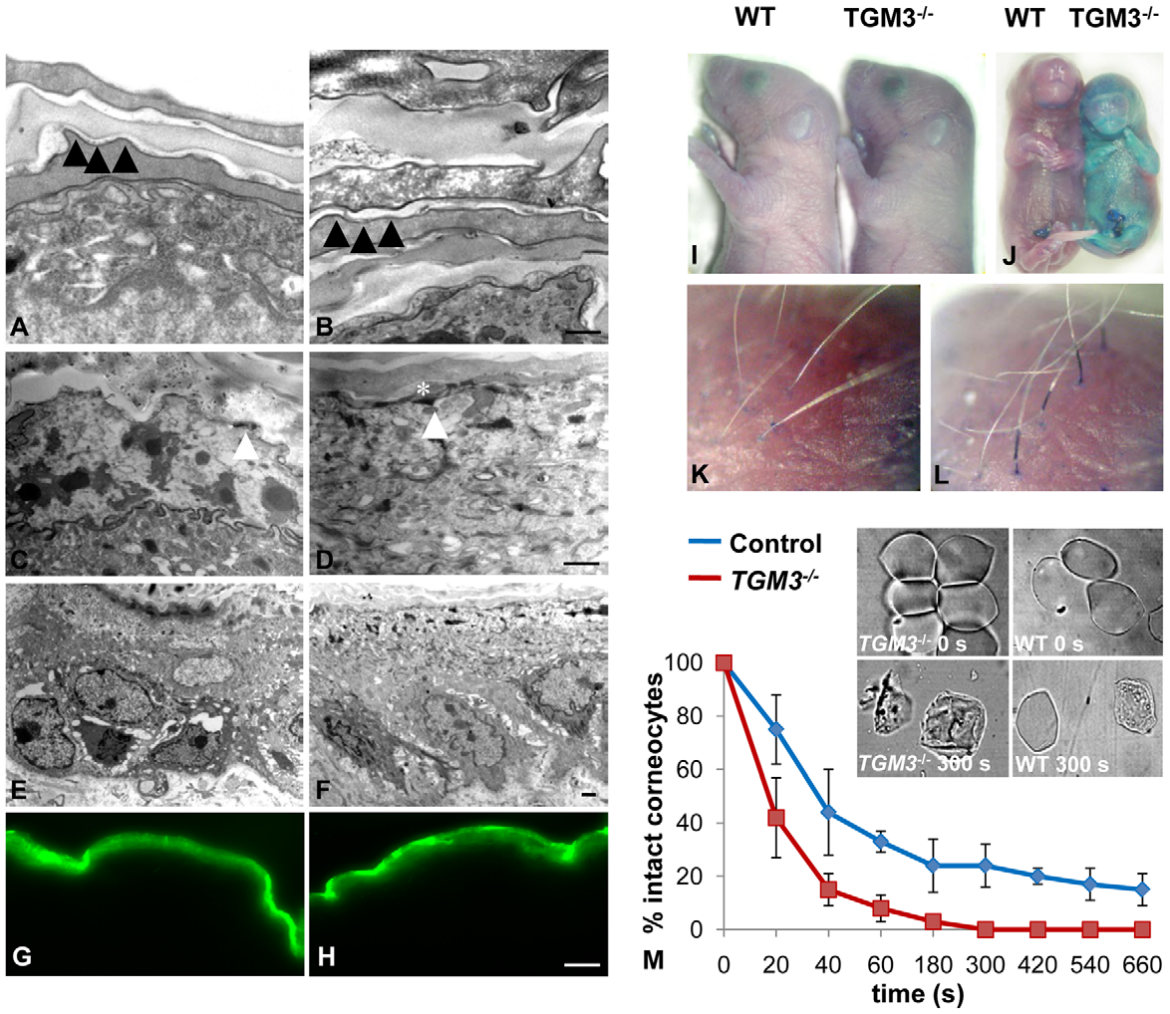
Barrier function of the skin in *TGM3^−/−^* mice. Transmission electron microscopy of skin from *TGM3* ^−/−^ (A, C and E) and wild type (B, D and F) 4 week old animals. Cornified cell envelopes (closed arrowheads in A and B) and keratohyalin granules (*) were visible in mice of both genotypes and in some sections. The latter could be seen coalescing with the forming cornified envelope of granular layer keratinocytes (open arrowheads in C and D). The cells of the *stratum corneum* in both wild type and *TGM3^−/−^* animals consisted of a defined cell envelope surrounding compact, electron-dense cytoplasm containing condensed tonofibrils. Lucifer yellow failed to penetrate through the cornified envelope in either the newborn *TGM3* ^−/−^ (G) or wild type skin (H). (scale bars B 0.5 µm, D 7 µm, F and H 50 µm). At birth there was no retention of toluidine blue dye in the skin of either *TGM3^−/−^* or wild type neonates (I). Dye was retained in the basal region of the whiskers in *TGM3* ^−/−^ (L) neonates, a finding not seen in wild type mice (K). While formation of the epithelial barrier had occurred in control mice at E17.5, toluidine blue penetrated the skin of *TGM3* ^−/−^ litter mates (J). Sonication of corneocytes isolated from skin biopsy punches for various times revealed that *TGM3^−/−^* corneocytes were more susceptible to lysis (M). The number shown is that of intact corneocytes remaining as a percentage of those initially isolated (n = 4).

**Figure 5:**
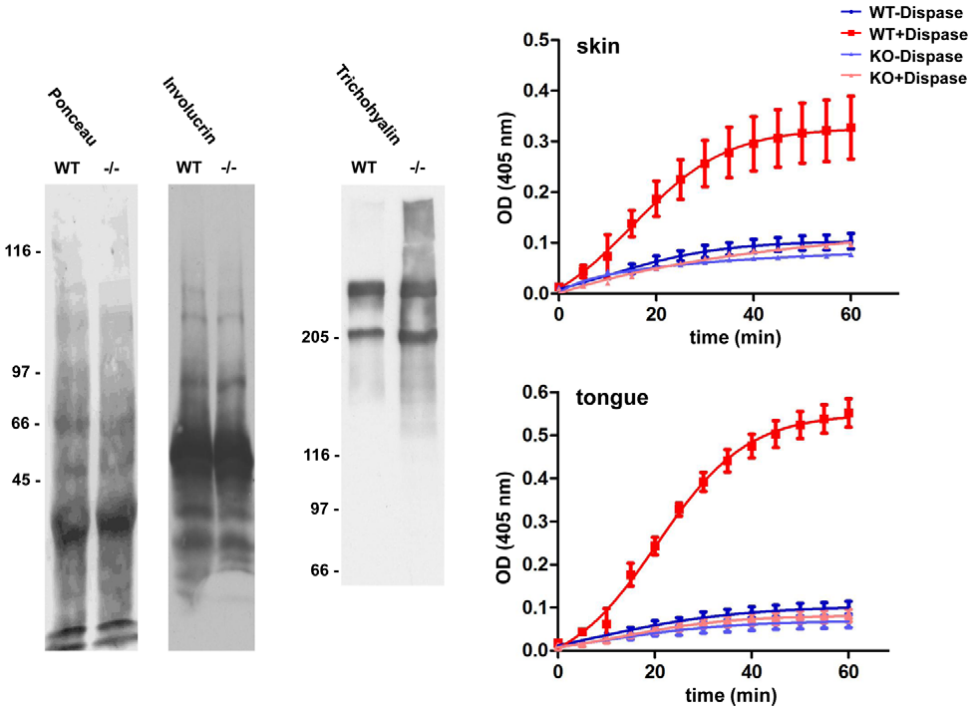
Protein extractability and transglutaminase activity in *TGM3^−/−^* tissue. Proteins from tissue extracts of *TGM3* ^−/−^ and control skin were separated on an 8% SDS-PAGE and transferred to nitrocellulose. Probing with antibodies against involucrin revealed no alteration between the two genotypes, however the minor skin component trichohyalin showed an increased extractability (A). Protein extracts from wild type and *TGM3* ^−/−^ skin and tongue were analyzed for biotin-cadaverin incorporation into NN′dimethylcasein, coated onto microtitre plates. Zymogen activation of the protein extract with dispase induced activity only in extracts from wild type mice (B).

Mutations leading to loss of TGase 1 activity cause severe changes in the cornified layer and the lipid envelope. To test whether TGase 3 could have a physiological function in the cornified layer, we assayed the structure and function of the epithelial barrier. We investigated transepidermal water loss in newborn mice with a Tewameter and also the passive inward diffusion of Lucifer yellow dye through the *stratum corneum* (Fig. 4G and H). Both of these measurements showed no change in the absence of TGase 3 either in adult or neonatal mice. During gestation TGase 3 is first expressed by the periderm at E12.5 and then is seen in the developing epidermis before E16.5, a time point when the epidermal barrier function first becomes evident [26]. Toluidine blue staining was used to analyze the barrier function. Neither wild type nor *TGM3* ^−/−^ perinatal mice showed toluidine blue retention in the epidermis (Fig. 4I) but dye was maintained by a portion of the whisker directly where it emerged from the skin (Fig. 4L). The formation of a water impermeable barrier occurs from day E16.5 and skin of E17.5 control embryos retained no toluidine blue. In contrast, *TGM3* mutants retained the pigment, particularly over the abdominal regions, suggesting a delay in formation of the skin barrier (Fig. 4J). These findings indicate that, while there may be retardation in its development, there is little change in epidermal barrier function at birth or later. To verify this, skin from the ear, back and belly of female *TGM3* ^−/−^ and wild type litter mates was analyzed by electron microscopy. A normal cornified envelope was present upon corneocytes (Fig. 4A and B) and keratohyalin granules were detected in both mutant and control skin in the upper granular layers, in some cases coalescing with the developing cornified envelope (Fig. 4C to F). Corneocytes isolated from the pinna appeared morphologically comparable in mutant and wild type mice and were obtained in similar yields. To assay for stability, corneocytes were kept under reducing conditions and subjected to mild sonication. Those from wild type mice showed a greater resistance to this treatment, with over 25% of corneocytes remaining intact after 5 minutes sonication, a time when all mutant cells had been disrupted (Fig. 4M). This suggested that despite a normal postnatal skin barrier there where changes in corneocyte stability. Covalent cross-links between keratins, *i.e.* disulfide bridges and Nε-(γ-glutamyl)lysine isopeptide bonds, are crucial for epidermal integrity. To see if there was an obvious alteration in stability of the skin protein network in *TGM3* mutants, skin from four week old mice was extracted under reducing conditions and subjected to immunoblotting for known TGase 3 substrates. No difference was seen in the extractability of epidermal keratins (results not shown) or keratin related proteins such as involucrin (Fig. 5A). Nevertheless, there was a slight increase in the extractability of trichohyalin, a minor component of the epidermis (Fig. 5A). Given that barrier formation was delayed, we tested the developing epidermis at E16.5. Again we found no difference in the extractability of either keratins or involucrin from the forming skin (results not shown). To test for alterations in transglutaminase enzyme activity in epithelia, we extracted soluble transglutaminase from the epidermis and tongue epithelium. There was very little activity without activation by dispase in wild type mice (Fig. 5B) and no difference between dispase treated and untreated extracts from *TGM3^−/−^* mice. This shows that almost all soluble transglutaminase activity in these epithelia is contributed by TGase 3.

### TGase 3 does not alter the wound healing rate in epithelial wounds

**Figure 6:**
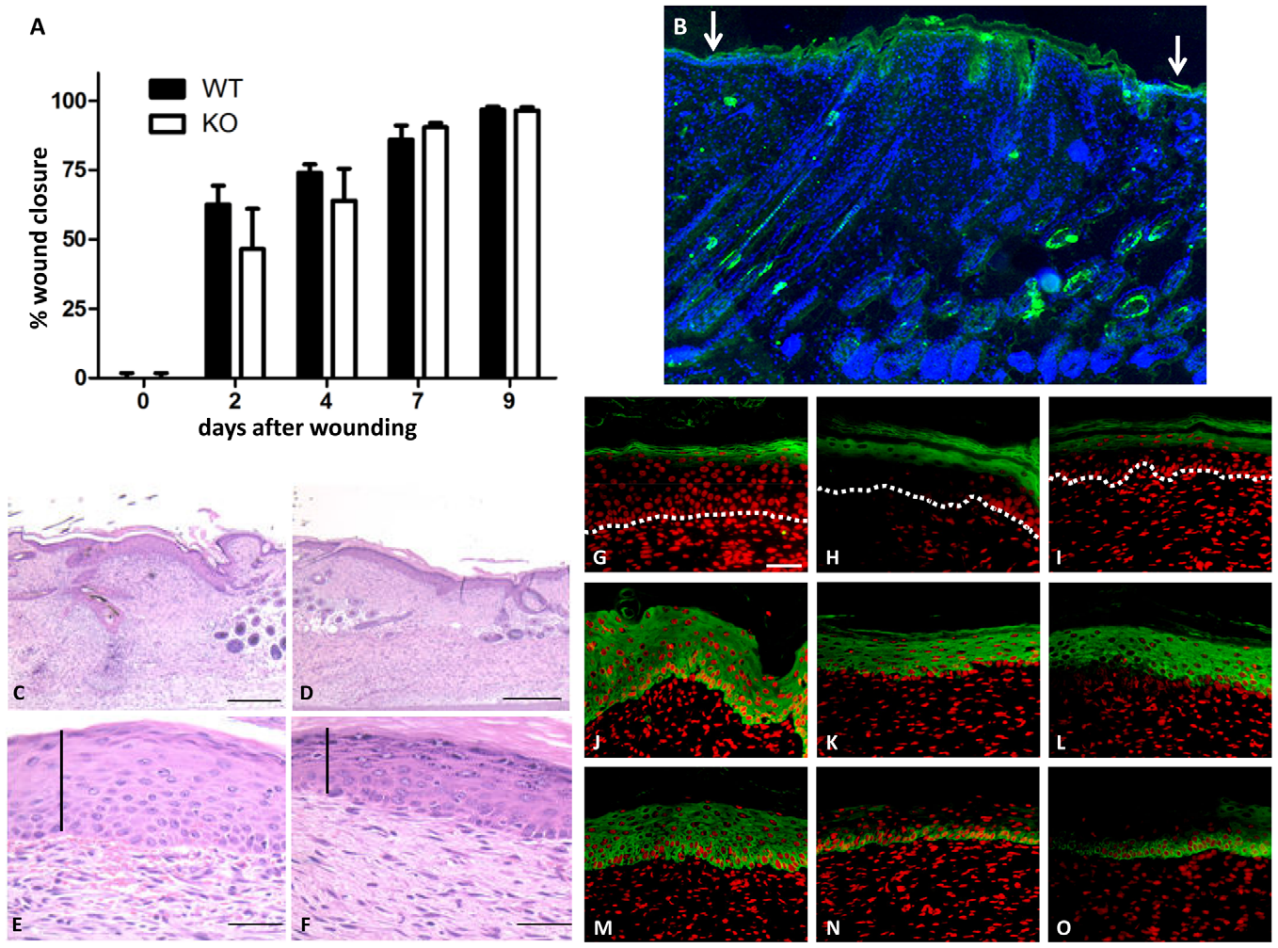
Wound healing in *TGM3^−/−^* skin. The rate of wound closure in *TGM3* ^−/−^ mice was not different compared to control animals (A) (n = 9), despite an increase in TGase 3 expression in healing epidermis (B). At day 9 post wounding the thickness of the epidermal layer in the wounded region was greater in wild type skin (C, E) when compared to mutant skin (D, F). Staining of healing skin with keratinocyte differentiation markers in wild type (G, J, M) and *TGM3^−/−^* (H, I, K, L, N, O) animals demonstrated that while there was no change in the expression pattern of the keratinocyte markers loricrin (G–I) or keratin10 (J–L) between the mouse lines, keratin14 (M–O) already showed a more restricted staining pattern reminiscent of unwounded skin. (scale bars C, D 400 µm, E–G 100 µm)

Transglutaminases are upregulated after wounding and are implicated in healing events [27]–[30]. To discover if TGase 3 is important in this process, full thickness wounds were excised with a 4 mm diameter biopsy punch. While TGase 3 staining is increased in the healing wild type epidermis (Fig. 6B), the rate of wound closure was unaltered between mutant and control (Fig. 6A). We then studied if the expression of keratinocyte markers in the healing wound was changed. While loricrin (Fig. 6G–I) and keratin10 (Fig. 6J–L) show the expected widened expression pattern seen in healing epidermis, keratin14 appears more restricted in the wounds of mutant mice (Fig. 6N and O) than in control animals (Fig. 6M), with an expression pattern closer to mature epidermis. Further, the healing epidermis was thinner in the mutant animals and appeared more differentiated (Fig. 6C–F).

### TGase 3 is required for proper hair development

**Figure 7:**
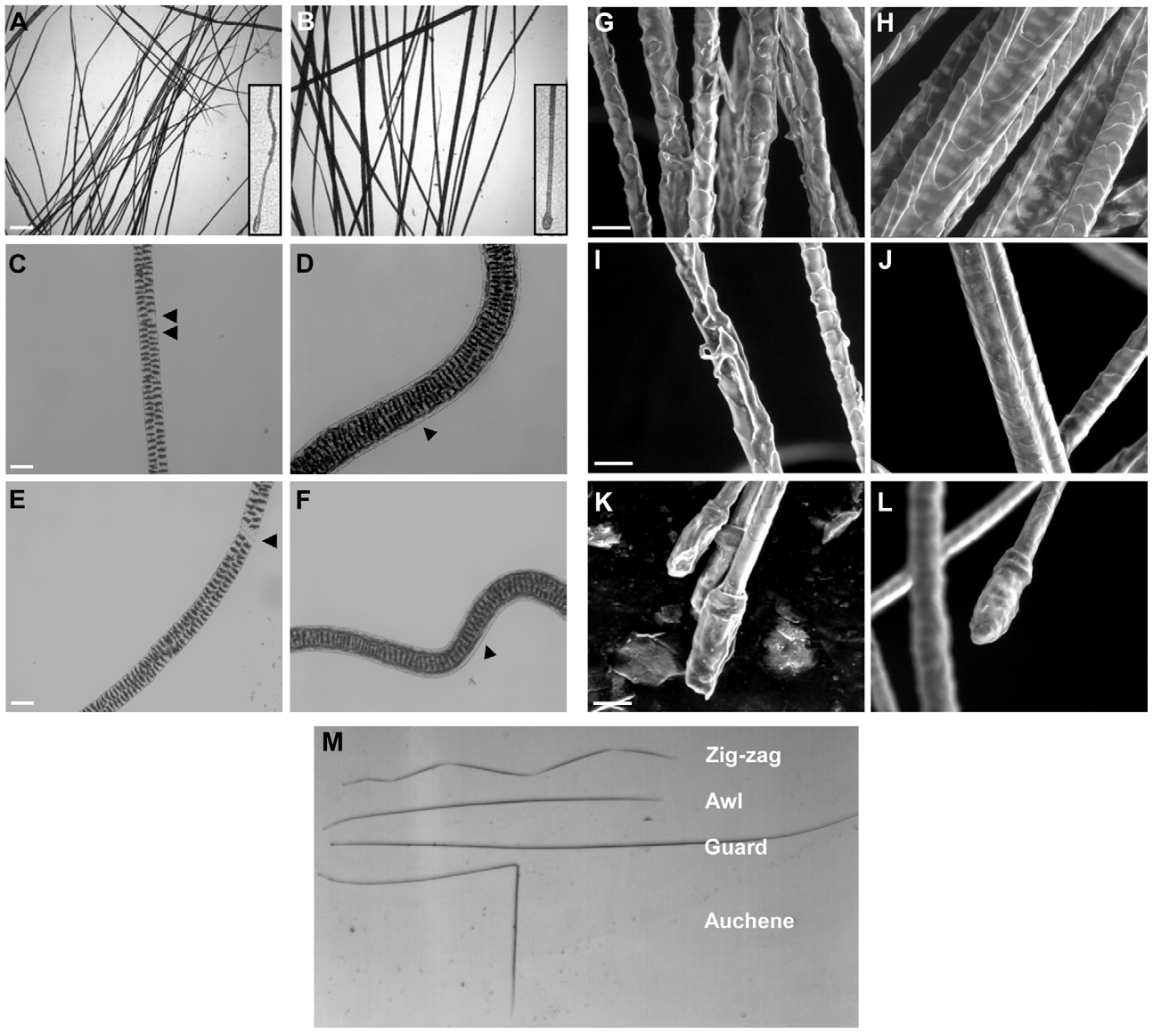
Morphological changes in pelage hair lacking TGase 3. Whole-mount light microscopy of *TGM3* ^−/−^ (A) and wild type (B) hair (scale bar 10 µm). Inserts show higher magnification of lower regions of the hairs. Hair incubated overnight at 65°C with agitation in 2% SDS and 20 mM dithiothreitol (C–F) *TGM3* ^−/−^ (C, E) and control hairs (D, F). Arrowheads indicate regions lacking or retaining the cuticle cells in the mutant and control hairs, respectively. Scanning electron microscopy of mutant (G, I) and control (H, J) hair shafts shows severe distortion of the hair, in particular the cuticle, in the absence of TGase 3. In contrast, the roots of the mutant hairs (K) appear similar to the wild type ones (L) (scale bar 20 µm). Each of the four main pelage hair types was present in the mutant animals (M).

**Figure 8:**
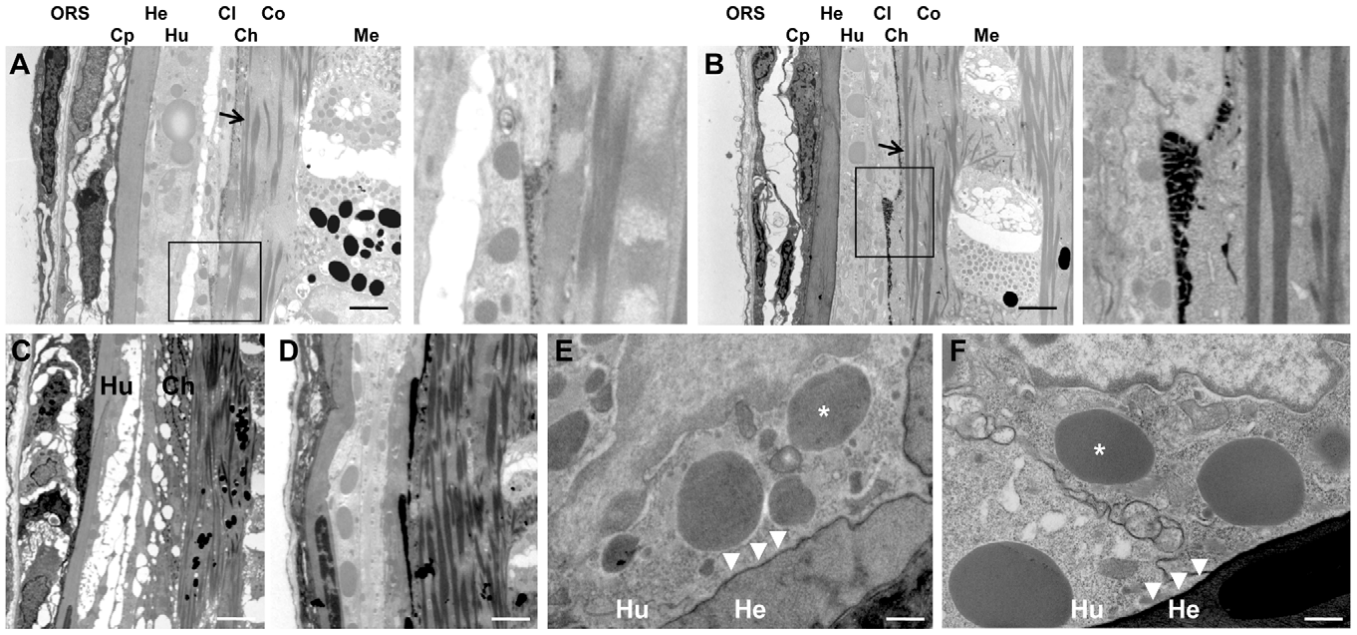
Transmission electron microscopy of sections through the hair follicle of *TGM3^−/−^* (A, C) and wild type hair (B, D) (scale bar 10 µm). Arrows show the keratin filaments. The cuticle layer is distorted in the mutant (magnified region), and disruption of the Huxley's layer is evident. Trichohyalin droplets (*) are seen as non-membrane-bound inclusions, in Huxley's layer of the IRS in both *TGM3^−/−^* (E) and control mice (F) and cornification occurs on the Henle's layer (arrow heads) (scale bar 2 µm). ORS-outer root sheath, Cp-companion layer, He-Henle's layer, Hu-Huxley's layer, Cl-cuticle of inner root sheath, Ch-hair cuticle, Co-hair cortex, Me-hair medulla.

*TGM3* ^−/−^ mice could be identified by the second day after birth due to markedly irregular whiskers (Fig. 2E–F). Even though the whiskers were often later lost, the number, positioning and layering of the whisker follicles was normal. Microscopy showed that the vibrissae were twisted and thinner than in controls. Pelage and tail hair also showed a wavy pattern. Interestingly, this was most obvious in the first four weeks (Fig. 2B–D). While adult mice had grossly normal looking coat hair, their whiskers, when retained, continued to be irregular. Nevertheless, the mutant hair in adult mice appeared irregular with an increased number of uneven, thinner hairs, displaying irregular torsions when looked at under the microscope (Fig. 7A). To verify presence and numbers of the four hair types (auchene, zig-zag, guard and awl), hairs were plucked and typed. While all hair types were present (Fig. 7M), the numbers of zig-zag type hairs were decreased (p = 0.028) when compared to control litter mates at 4 months age (78% in control, 63% in *TGM3^−/−^* mice). Further, they appeared brittle with damaged hairs present and the thinner hairs often broke during manipulation in the counting process. Despite this, baldness was not generally observed. Hairs were then examined by scanning electron microscopy. This showed an irregular twisting of many hairs in the mutant with the even groove, found in normal hairs, often being absent or highly deformed (Fig. 7G–J). The regular and overlapping shingle-like structure of the cuticular scales, while maintained in some *TGM3^−/−^* hairs, was often distorted. In some cases small cracks occurred in the cuticle, while in others scales could be seen lifting from the underlying cortex (Fig. 7G, I). In agreement with the finding that the mutant mice did not show alopecia, tape stripping suggested the hair bulb was no less well attached to the follicle and scanning electron microscopy of the anagen hair bulb indicated no differences between *TGM3^−/−^* and control mice (Fig. 7K, L). To determine if the integrity of the hair was compromised, plucked hairs were treated overnight with detergent and a reducing agent to break any disulfide bonds. In wild type hair the overall structure was maintained, with the cuticle being retained and the structure of the medulla remaining intact (Fig. 7D, F). In contrast, mutant hair showed long stretches where the cuticle was lost and in places the medulla distorted (Fig. 7C, E). We then examined the pelage hair and whiskers by transmission electron microscopy. The usual layers in the hair shaft and follicle were evident in the mutant hairs (Fig. 8A, B). Both the outer and inner root sheath were present, while the amorphous Henley's layer appeared normal with an evident cornified cell membrane (Fig. 8E, F). In contrast, the Huxley's layer, containing vacuoles filled with trichohyalin, was often torn or distorted (Fig. 8A, C). Further, the cuticle of the hair was abnormal, the outer exocuticular layer being irregular and having a reduced number and electron density of granules underlying the cell membrane. In the cortex and medulla, keratin filaments were obvious in the mid-shaft region but in mutant hair the keratin filaments in cortex cells were often shorter and less regular.

### Biochemical defects in cross-linking of the hair

**Figure 9:**
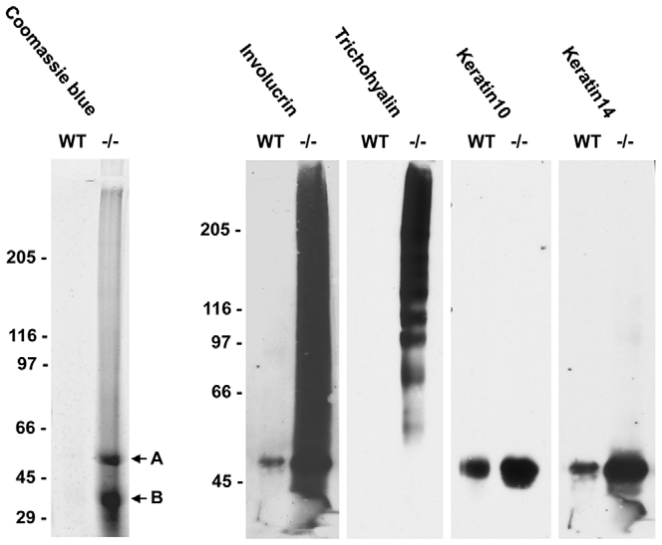
Protein extractability in *TGM3^−/−^* hair. Proteins were extracted from wild type and *TGM3^−/−^* hair with 2% SDS and 5% β-mercaptoethanol over night prior to SDS-PAGE separation and transferred to nitrocellulose. Membranes were probed with polyclonal antibodies against involucrin, trichohyalin, keratin10 and keratin14. Two prominent bands observed in the Coomassie stained SDS-PAGE of *TGM3^−/−^* protein lysates were identified by tryptic peptide mass fingerprinting as keratin6hf (A) and keratin17 (B).

We then questioned whether there were definable defects in cross-linking in the hair due to the absence of TGase 3. Proteins were extracted under reducing conditions from equal amounts of plucked hair of 14 days and 4 months old mice. At both ages, far more protein was isolated from mutant hair (Fig. 9 and results not shown). The extracts were then submitted to SDS-PAGE and immunoblotting and probed for known substrates of TGase 3. In all cases there was a marked increase in extractability, but this was less pronounced for keratin10 and 14, which are present in the inner and outer root sheath of the hair follicle, respectively. A dramatic change was seen in the two keratin associated proteins, involucrin and trichohyalin, the former of which was only weakly extractable and the latter was not extractable from control hair. For both a strong signal was observed with the main protein bands being accompanied by an immunoreactive smear, suggesting partial cross-linking of these proteins into a matrix. The SDS-PAGE separation of *TGM3^−/−^* hair extracts also revealed two prominent protein bands upon Coomassie blue staining which were absent from control extracts (Fig. 9). These bands were cut from the gel, in-gel digested with trypsin and the peptides obtained analyzed by MALDI-TOF mass spectrometry. This allowed the identification of keratin6hf and keratin17, two keratins present in both the medulla of the hair and the companion layer of the outer root sheath of the hair follicle.

## Discussion

Despite the widespread expression of TGase 3 [15], its absence in the mouse does not cause severe malformation. Further, *TGM3^−/−^* mice are fertile and produce litters of the expected size. Of the nine transglutaminases [8], six are expressed in epithelia [16], [31] but only TGase 1, TGase 3 and TGase 5 are considered participants in cornified envelope assembly [10]. While the main proteins of the cornified envelope are all cross-linked by isopeptide bonds, the different transglutaminases show a high specificity for certain glutamine and lysine residues in these substrates [8], [9]. This, along with the timing of expression of the transglutaminases in the differentiation pathway and position within the cell (TGase 1 at the plasma membrane, TGase 3 in the cytosol and TGase 5 upon the actin cytoskeleton), led to the hypothesis that the enzymes act in sequence to produce the cornified envelope [6], [10]. TGase 3 is believed to have a role in intermolecular cross-linking of cytoplasmic structural components into small complexes which are integrated into the immature cortical protein complex by TGase 1. However, our studies failed to show major defects in the interfollicular epidermis. Indeed, electron microscopy demonstrated distinct cornified envelopes and there was no increase in the solubility of proteins normally present in these structures. Also, tape stripping did not demonstrate a change in epidermal integrity, but isolated corneocytes from these mice appear more fragile. Presumably, in *TGM3^−/−^* mice the cross-linked network is stable enough to prevent extraction of proteins, but severe stress does demonstrate that its loss is not fully compensated for. Gene targeting of many of the skin's structural components has revealed redundancy or compensatory mechanisms in other aspects of the cornified envelope [21], [22], [32]. Loss of loricrin, a major constituent of the *stratum corneum* and a substrate for TGase 3, does not alter epidermal barrier function postnatally [22]. Permeability changes occur in mouse embryonic skin, starting abruptly at E16 and being complete by E17 [33]. Loricrin expression correlates with this change and as *TGM3* mutants, *Lor^−/−^* embryos have a delay in skin barrier development with isolated corneocytes being more easily fragmented by sonication. It is possible that TGase 3 cross-linking has a more significant role before birth as it is expressed in the periderm from E12.5 [26], while TGase 1 is only fully expressed by E17.5 [34], even though we were unable to demonstrate an increased solubility of proteins from the developing skin of *TGM3^−/−^* mice at E16.5. TGase 3 is also present in other tissues including brain, heart and placenta [6], [15], [26]. Although there are no obvious changes in these tissues, slight defects could result in a developmental delay, which may be an alternative cause of the late barrier development. The murine hair shaft consists of the medulla and cortex covered by a single layer of cuticle cells, both of which have been shown to have TGase 3 activity [35]. It is enveloped by the inner root sheath, consisting of its own cuticle, which interdigitates with the hair, the Huxley's and the Henle's layer. The inner root sheath stretches from the bulb to the mid-isthmus where it degenerates. It is surrounded by the outer root sheath, which is continuous with the interfollicular epidermis and encases the entire follicle. Numerous cell types in the hair show cornified envelope formation and the inner root sheath has a very high content of isopeptide cross-links (about 1 residue in 30) [24]. As in the skin, transglutaminases are expressed in a manner dependent on the cell type and on their differentiation stage, with TGase 2 being present in the bulb region, TGase 1 and then TGase 3 in later inner root sheath development [24]. Mechanically strong epithelia express high levels of trichohyalin, an intermediate filament associated protein which has a particularly high content of glutamine and lysine residues (23%) [36]. Trichohyalin is co-expressed with TGase 3 in other stratified epithelia, such as the filiform ridges of the tongue and the keratinized stomach of the rodent [37]. Even so, we failed to find any defects in these structures in *TGM*3^−/−^ mice. Trichohyalin is also abundant in the medulla and in the inner root sheath of developing hair where it is cross-linked to keratin filaments [25]. After terminal differentiation trichohyalin cannot usually be extracted by conventional means and its release from *TGM3* ^−/−^ hair agrees with previous results suggesting that trichohyalin cross-linking depends on TGase 3 rather than TGase 1, despite the latter enzyme being present in the inner root sheath [24]. Further, *in vitro* studies showed that TGase 3 has a high preference for trichohyalin and keratin filaments as substrates [25], [38], [39]. Human data suggests that TGase 3 is the predominant and possibly only transglutaminase present in the hair cuticle and cortex, which also develop a cornified envelope [40], [41]. Electron microscopy showed that many hairs in *TGM3^−/−^* mice had a highly distorted cuticle, which was poorly retained upon the underlying cortex. Mild heating of hair in a reducing solvent led to loss of the cuticle. It is not known whether this is caused by breakage of bonds between the cuticle and the cortex or breakdown of the cuticle cells itself. Transmission electron microscopy showed that the subcuticular zone failed to form normally in the *TGM3^−/−^* animals suggesting that the hair cuticular cells are directly compromised. Further, the Huxley's layer which has a high expression of transglutaminases, including TGase 3 [24] appears fragile. Keratin filaments are cross-linked into the cornified envelope by both disulfide and isopeptide bonds. Indeed, many type 2 keratins are TGase 3 substrates *in vitro* [39] where a conserved lysine is utilized. Keratin6hf is present in the medulla and matrix of the hair shaft [42], co-localizing with keratin17 [43], and it appears that neither the other keratin6 isoforms nor the closely related keratin5 are expressed here. Our results provide evidence that keratin6hf and keratin17 form filaments *in vivo*, in agreement with the co-localizing seen by confocal microscopy [42]. The high solubility of these keratins and of trichohyalin in *TGM3* ^−/−^ mice strongly suggests that they form a complex stabilized by isopeptide bonds. Mutations in either keratin6hf or keratin17 are related to hair defects. *K17^−/−^* mice show alopecia with defects in primary hair growth when kept on a C57/Bl6 background [44]. The hair in these mice is extremely fragile with increased apoptosis in the matrix of the hair and follicle degeneration. Interestingly, as in *TGM3* ^−/−^ mice, the phenotype improves after the first postnatal hair cycle. This finding and the genetic strain specificity appears to be caused by a compensatory increase in keratin16 levels [45]. Curiously, in both the *K17* ^−/−^ and *TGM3* ^−/−^ mice there is no nail defect despite both genes being expressed in the nail bed, even though missense mutations in keratin17 are related to type 2 *pachyonychia congenita*, which presents with severe nail dystrophy [44] and *pili torti* [46]. Though severe twisting in the hair shaft, present in human *pili torti*, was generally not observed in these mice, some hairs did show constrictions and torsions. Other human recessive forms of *pili torti* which improve with age have been described [47] and it is possible that these are related to changes in TGase 3 or its substrates. Indeed, recent findings have shown that variation in the trichohyalin gene associates with the straight hair phenotype in man [48]. Humans with *lamellar ichthyosis* have severe hair abnormalities [49] and skingrafted from murine *TGM1* ^−/−^ neonates develop abnormal hair follicles [50]. However, it is not known whether this is caused by specific changes in hair development or by the overall disruption of the grafted tissue. It would appear that TGase 1 is crucial for the development of the normal hair follicle, while TGase 3 plays a more subtle role, particularly in the formation of the hair cuticle. As in the skin, TGase 3 expression occurs later in hair maturation than TGase 1 [24], and it may act to reinforce the developing cornified envelope initiated by TGase 1, by introducing trichohyalin and keratin17/keratin6hf containing intermediate filaments into the cornified envelope. There is much circumstantial evidence of a role for transglutaminases in wound healing. Surprisingly, there was little change in the rate of wound closure upon the loss of TGase 3. In immature wounds, keratinocytes express a wide spectrum of keratins, which become limited as the tissue matures. Post wounding *TGM3^−/−^* skin more rapidly developed a keratin expression pattern closer to that of unwounded skin when compared to controls. Further, there was an obviously thinner epidermal layer at day 9 after wounding in the mutants, despite no statistical change in cell number when compared to control wounds. This suggests the “activated” keratinocytes seen in wound healing changed in the absence of TGase 3. These cells produce increased levels of transglutaminases and growth factors [51]. TGase 3, like factor XIII and TGase 2, is released from cells [16], [39] and may play a role in TGFβ activation [52], fibronectin signaling [53], and cross-linking of the extracellular matrix [29], [54]. Transglutaminases are up-regulated in migrating keratinocytes at the wound edges [27]. Interestingly, keratin6hf and keratin17, which are substrates for TGase 3, are also rapidly up-regulated here, with a concomitant reduction in keratin1 and 10 [42]. In conclusion, despite a widespread expression and much circumstantial evidence as to its importance in the formation of the cornified envelope, it appears that loss of TGase 3 is largely compensated in stratified epithelia. TGase 3 is highly significant in hair development and in particular the cuticle, where it appears to have a unique role in stabilizing the trichohyalin network.

## Materials and Methods

### Ethics statement

Animal studies were performed in accordance with institutional guidelines. The protocol was approved by the District Council of Cologne, North Rhine-Westphalia, Germany (Permit Number: 52.203.2-K46, 22/02).

### Generation of *TGM3^−/−^* mice

The 5′ flanking arm of the targeting construct, a 3.1 kb fragment containing exon 5, intron 5 and a portion of exon 6 of the *TGM3* gene was amplified by PCR from genomic mouse DNA with SacII and NheI/SalI restriction sites integrated into the 5′ and 3′ primers, respectively. The 3.2 kb 3′ region directly flanking exon 6 and containing exon 7 was also amplified by PCR with SalI restriction sites included in the primers. These fragments were cloned into pBluescript and a neomycin-resistance gene expression cassette was then inserted into the NheI site. The targeting vector was linearized with ClaI and electroporated into E14 (subclone IB10) mouse ES cells. After G418 (GIBCO) selection, clones were analyzed for homologous recombination by Southern blotting, hybridizing with external probes both at the 3′ and 5′ end as well as an internal probe. ES cells carrying the disrupted allele were microinjected into C57/BL6 blastocysts. Resulting chimeric mice were bred to derive animals heterozygote for the *TGM3* mutation. These were subsequently either interbred to produce homozygous mice or repeatedly crossed onto a C57/BL6 background.

### Histology and immunofluorescence staining of tissues

For histology, tissues were fixed in 4% paraformaldehyde, embedded in paraffin and 6 µm sections cut. Sections were stained with haematoxylin and eosin. For immunofluorescence microscopy, either unfixed tissue, embedded in Tissue Tec (Sakura, Japan) and snap frozen in liquid nitrogen or paraffin embedded tissue was sectioned. After blocking, sections were incubated for 1 h at RT with rabbit polyclonal antibodies against loricrin, involucrin, filaggrin, keratin1, keratin10, keratin14 (Covance Research Products), and trichohyalin (a kind gift from Dr. Edward O'Keefe, University of North Carolina, USA), mouse monoclonal antibodies against TGase 1 (Paesel+Lorei) or rat monoclonal antibodies against TGase 2 (Neomarkers) and TGase 3 (a kind gift from Dr. Kiyotaka Hitomi, Nagoya University, Japan). Labeling was detected with goat antibodies against rabbit, mouse or rat immunoglubulins conjugated to Cy3 or Alexa 488. Sections were examined with an Axiophot microscope (Zeiss).

### Immunoblotting and protein detection

Tissues of *TGM3* mutant and control mice were homogenized in cold lysis buffer (20 mM Hepes, 100 mM NaCl, 1 mM phenylmethylsulfonyl fluoride, 1% Triton X-100 and 0.1% SDS), centrifuged at 16.000× g for 30 min at 4°C and the supernatant was removed. For the extraction of hair protein, hairs were plucked from 2 week and 4 month old mice, immersed in 2% SDS and 5% β-mercaptoethanol overnight at 4°C and after boiling for 5 min centrifuged at 16.000× g for 30 min at 4°C. Proteins in the supernatants were separated on an 8% SDS polyacrylamide gel and either stained with Coomassie brilliant blue or transferred to nitrocellulose. After blocking either with 5% nonfat milk or with 1% casein dissolved in TBS, the nitrocellulose membranes were incubated with primary antibodies. These were rabbit polyclonal antibodies against TGase 3 [16], loricrin, involucrin, filaggrin, keratin1, keratin10, keratin14, or trichohyalin, and mouse monoclonal antibodies against TGase 3 (for sources see the section above). Mouse monoclonal antibodies were diluted in 1% casein/TBS while rabbit polyclonal antibodies were diluted in 5% nonfat milk/TBS. After probing with the primary antibodies for 1 h at RT and washing, membranes were incubated with horseradish peroxidase-conjugated swine antibodies against rabbit or mouse IgG (Dako) followed by chemiluminescence detection (ECL, Amersham). For identification of proteins, bands from Coomassie brilliant blue stained gels were excised, in-gel digested with trypsin and analyzed in a Bruker Reflex IV MALDI-TOF mass spectrometer. The identity was determined from the peptide masses using the Mascot search engine.

### Transglutaminase activity

For transglutaminase activity assays, NN'dimethylcasein was coated onto microtitre plates, which were then blocked with BSA. Skin proteins were extracted by homogenization in 0.1 M Tris-HCl, pH 8.0 followed by clarification via centrifugation. In some cases dispase was added to activate zymogen forms of the enzyme. The supernatant was placed in the wells in the presence of 25 mM CaCl_2_ and 0.5 mM biotin-cadaverin (Molecular Probes) as co-substrate. After incubation at 37°C, the reaction was stopped with EDTA. The wells were washed with 0.1 M Tris-HCl pH 8.0/0.01% Triton X-100 and incubated with Avidin-HRP for 1 h. After washing peroxidase activity was detected with ABTS (2,2′-azino-bis(3-ethylbenzthiazoline-6-sulphonic acid) in 0.03% H_2_O_2_ with the product measured OD at 405 nm.

### Hair Analysis

Hair integrity was analyzed by microscopy after incubating hair overnight at 65°C with agitation in 50 mM sodium phosphate buffer, pH 7.9, containing 2% SDS and 20 mM dithiothreitol to destroy disulfide bonds.

### Preparation of corneocytes

Corneocytes were isolated from 2 mm biopsy plugs from the ears of 4 week old female *TGM3* mutant and control mice. The tissue was heated at 95°C for 30 min in 25 mM DTT and 2% SDS in 10 mM Tris-HCl, pH 8.0, 1 mM EDTA, and subsequently centrifuged at 5000× g for 10 min. After discarding the supernatant and washing the pellets twice, corneocytes were suspended in 10 mM Tris-HCl, pH 8.0, containing 1 mM EDTA. The corneocytes where subjected to ultrasound for varying lengths of time using a cup horn sonicator (Misonix, Farmingdale). The corneocyte suspension was then examined for the presence of intact cells.

### Assays for barrier function

To analyze potential barrier defects, Lucifer yellow penetration assays were performed. 1 mM Lucifer yellow solution was placed upon the backs of new born mice. After 1 h the mice were killed and frozen sections were prepared. Penetration of the dye was analyzed using a fluorescence microscope. For toluidine blue staining, newborn mice were killed and then dehydrated by incubations (1 min each) in 25%, 50%, and 75% methanol/PBS followed by 1 min in 100% methanol. The cadavers were then rehydrated with the same series of methanol solutions (1 min incubations), washed in PBS, and stained for 1 min in 0.0125% toluidine blue (Fluka) dissolved in PBS. After destaining in PBS, the mice were photographed. Transepidermal water loss from the ventral and dorsal skin of adult mice was determined using a Tewameter as described previously [14].

### Wound Healing

For wound healing experiments *TGM3* deficient mice were back-crossed to C57/Bl6 mice for seven generations and then intercrossed to produce wild type and homozygous mutant litter mates. Wounds of 4 mm diameter were produced in anesthetized females by excising skin, the sub-cutaneous fat and the panniculus muscle with a biopsy punch (STIEFEL, Germany). Wounds were left uncovered and digitally photographed at the indicated time points. Macroscopic wound closure was determined by measuring the wound area and wound size was expressed as percentage of initial wound area. For histology, the complete wounds were excised with a margin of surrounding skin. Tissues were either fixed for 2 hrs in 4% paraformaldehyde before paraffin embedding or frozen unfixed in Tissue Tec. Serial sections were cut from the surrounding wound margin across the center of the wound towards the opposite wound edge in the caudocranial direction. The section with the largest wound diameter was defined as the wound center. Only sections from the wound middle were used for immunostaining. Antigen retrieval was performed by incubation with 0.1% trypsin for 10 min. at 37°C.

### Transmission and scanning electron microscopy

For transmission electron microscopy, 2 mm^3^ blocks of full thickness skin were fixed in 2.5% glutaraldehyde in 0.1 M cacodylate buffer. The specimens were then rinsed in 0.1 M cacodylate buffer plus 0.23 M sucrose and 2 mM CaCl_2_ at pH 7.2, postfixed in 2% osmium tetroxide in 0.1 M cacodylate buffer for 1 h, rinsed in buffer, stained with 2% aqueous uranyl actetate, dehydrated and embedded in Spurr's resin. 1 µm semi-thin sections were cut with glass knives on a Leica OMU3 ultramicrotome and stained with toluidine blue for light microscopy. Thin sections were cut, stained with lead citrate/uranyl acetate and visualized with a Hitachi H7000 electron microscope equipped with a SIS Megaview III digital camera. For scanning electron microscopy hairs were placed directly on an aluminium stub and viewed uncoated with a FEI Quanta 200 scanning electron microscope.

## Supporting Information

Figure S1 **Analysis of TGase 3 RNA expression upon insertion.** Exon 6 encodes the major part of the catalytic center, in particular the cysteine producing the substrate-enzyme intermediate. The selection cassette is inserted at base 790 and introduces a premature stop codon in any transcript formed containing exon 6 at amino acid 245 (murine TGase 3 containing a total of 693 residues). Further, the insertion of the 1600 bp (pgk-Neo-pA) is expected to destabilize this transcript. Should splicing occur around exon 6, not only would this remove the catalytic core region but exon 5 splicing to exons 7, 8, 9, 10 or 11 would generate a frame shift in the transcript. To verify the effect upon the *TGM3* transcript, mRNA was isolated from skin, reverse transcribed and assayed at either end of the native message. While the expected DNA fragments, 606 bp (amplified from exons 2 to 5) and 208 bp (exons 7 and 9), were obtained from both wild type and heterozygous skin, neither was seen from mutant skin suggesting instability of the transcript.
(TIF)

Figure S2 **Epithelial differentiation in** ***TGM3^−/−^*** **mice.** Histology showed no major changes in skin or other keratinizing compound squamous epithelia such as the oesophagus, filiform ridges of the tongue or the keratinized stomach (Fig. S2 A–H). We then studied expression of markers for skin keratinocyte differentiation, including keratin isoforms, filaggrin, loricrin and involucrin, all known *in vitro* substrates for TGase 3. None showed an altered expression pattern (Fig. S2 I–T). Upper panel, hematoxylin and eosin staining of epithelial tissues normally expressing TGase 3 in *TGM3^−/−^* (A–D) and wild type (E–H) mice. Ear (A, E), tongue (B, F), stomach (C, G) and back skin (D, H) (scale bars 100 µm). Lower panel, sections of back skin from *TGM3^−/−^* (I–N) and wild type (O–T) mice were incubated with polyclonal antibodies against markers for keratinocyte differentiation. Loricrin (I, O), keratin14 (J, P), keratin10 (K, Q), keratin1 (L, R), involucrin (M, S) and filaggrin (N, T) (scale bars 50 µm).
(TIF)

Figure S3 **Analysis of TGase 5 RNA expression upon insertion.** To check for any compensatory up-regulation in *TGM5* message, total RNA from skin was reverse transcribed by random priming and analyzed by qPCR. Relative expression values (2^ΔΔC(t)^) for *TGM5* were obtained by comparing it with the 18S amplification. No difference was observed between wild type (2.023, sd +/−0.411, n = 4) and *TGM3^−/−^* animals (2.01, sd +/−0.23, n = 4).
(TIF)
