# Supplementary figures and images for: Epidermal Transglutaminase (TGase 3) Is Required for Proper Hair Development, but Not the Formation of the Epidermal Barrier
Source: PLoS One. 2012 Apr 4;7(4):e34252. doi: 10.1371/journal.pone.0034252 (PMC3319564; doi:10.1371/journal.pone.0034252)

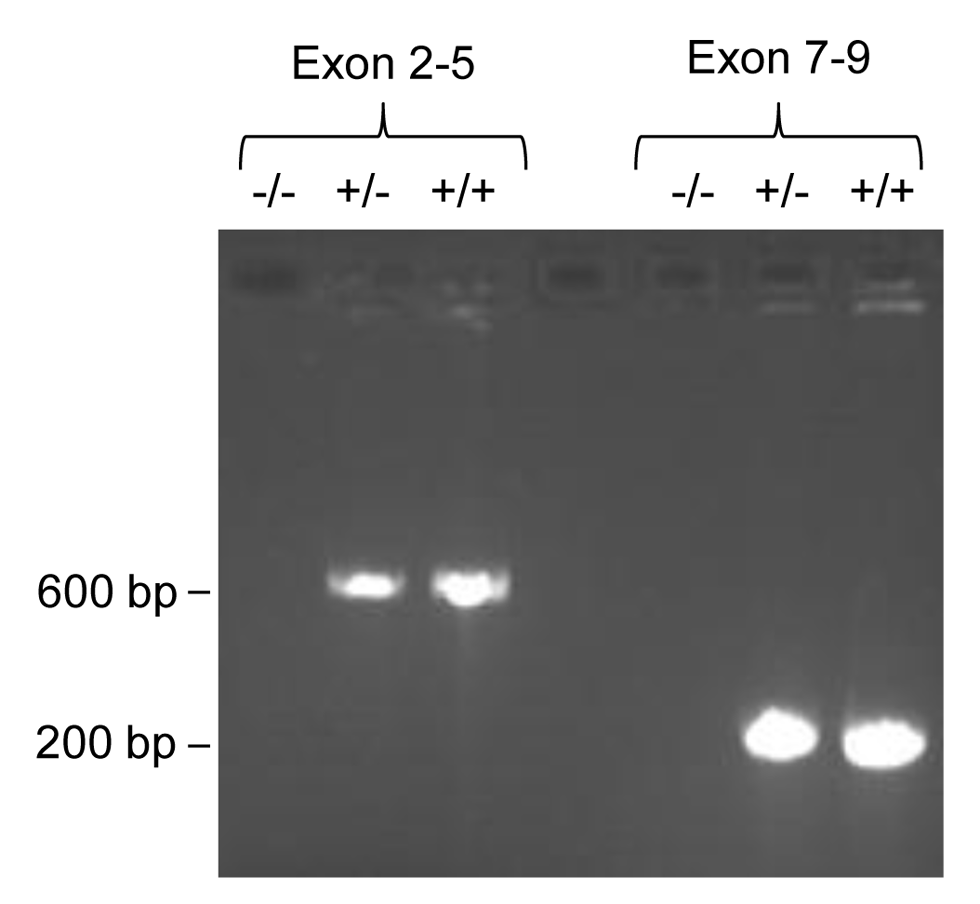

Supplement: Figure S1 — Analysis of TGase 3 RNA expression upon insertion. Exon 6 encodes the major part of the catalytic center, in particular the cysteine producing the substrate-enzyme intermediate. The selection cassette is inserted at base 790 and introduces a premature stop codon in any transcript formed containing exon 6 at amino acid 245 (murine TGase 3 containing a total of 693 residues). Further, the insertion of the 1600 bp (pgk-Neo-pA) is expected to destabilize this transcript. Should splicing occur around exon 6, not only would this remove the catalytic core region but exon 5 splicing to exons 7, 8, 9, 10 or 11 would generate a frame shift in the transcript. To verify the effect upon the TGM3 transcript, mRNA was isolated from skin, reverse transcribed and assayed at either end of the native message. While the expected DNA fragments, 606 bp (amplified from exons 2 to 5) and 208 bp (exons 7 and 9), were obtained from both wild type and heterozygous skin, neither was seen from mutant skin suggesting instability of the transcript. (TIF) [file pone.0034252.s001.tif]

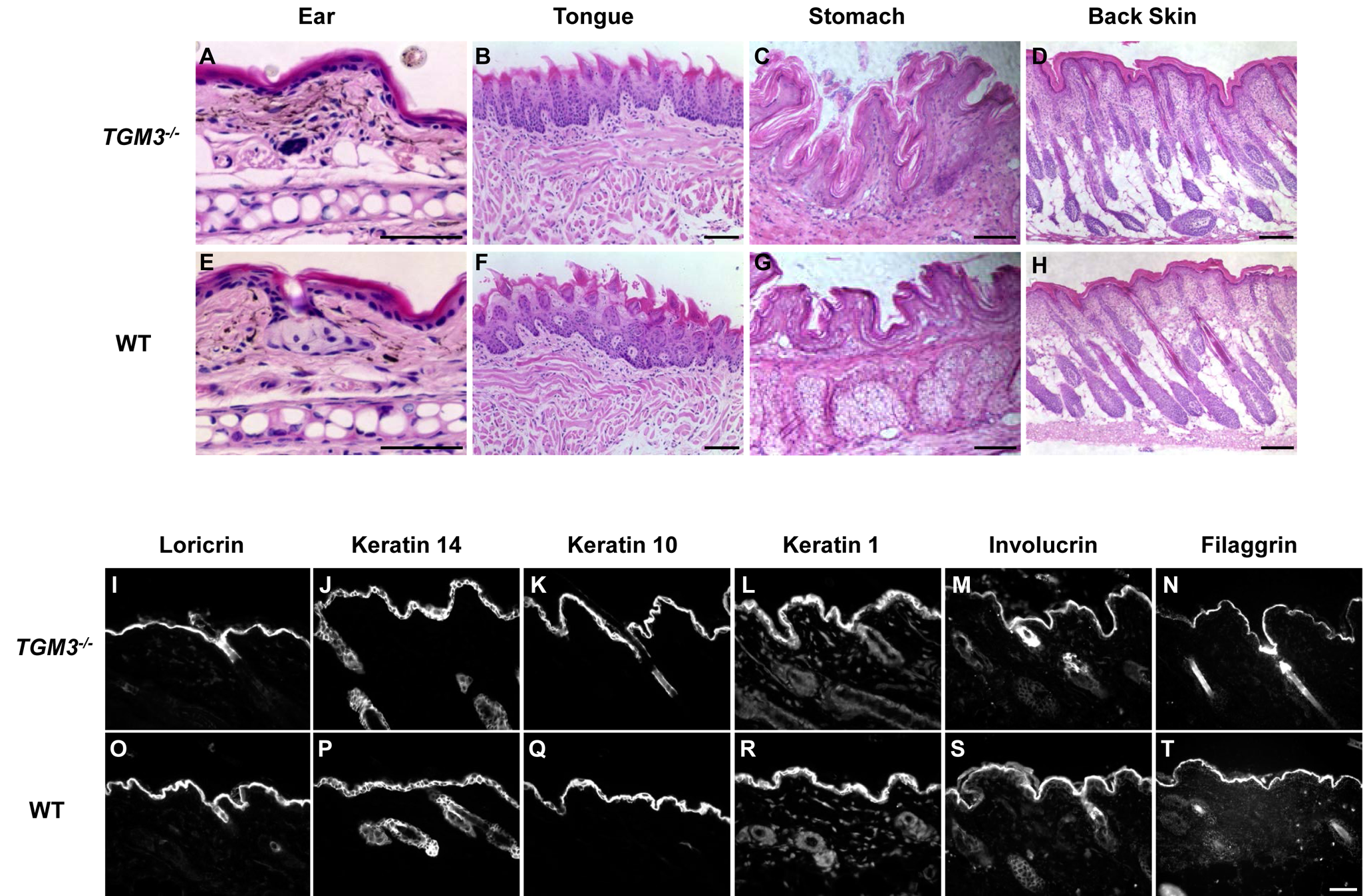

Supplement: Figure S2 — Epithelial differentiation in TGM3−/− mice. Histology showed no major changes in skin or other keratinizing compound squamous epithelia such as the oesophagus, filiform ridges of the tongue or the keratinized stomach (Fig. S2 A–H). We then studied expression of markers for skin keratinocyte differentiation, including keratin isoforms, filaggrin, loricrin and involucrin, all known in vitro substrates for TGase 3. None showed an altered expression pattern (Fig. S2 I–T). Upper panel, hematoxylin and eosin staining of epithelial tissues normally expressing TGase 3 in TGM3−/− (A–D) and wild type (E–H) mice. Ear (A, E), tongue (B, F), stomach (C, G) and back skin (D, H) (scale bars 100 µm). Lower panel, sections of back skin from TGM3−/− (I–N) and wild type (O–T) mice were incubated with polyclonal antibodies against markers for keratinocyte differentiation. Loricrin (I, O), keratin14 (J, P), keratin10 (K, Q), keratin1 (L, R), involucrin (M, S) and filaggrin (N, T) (scale bars 50 µm). (TIF) [file pone.0034252.s002.tif]

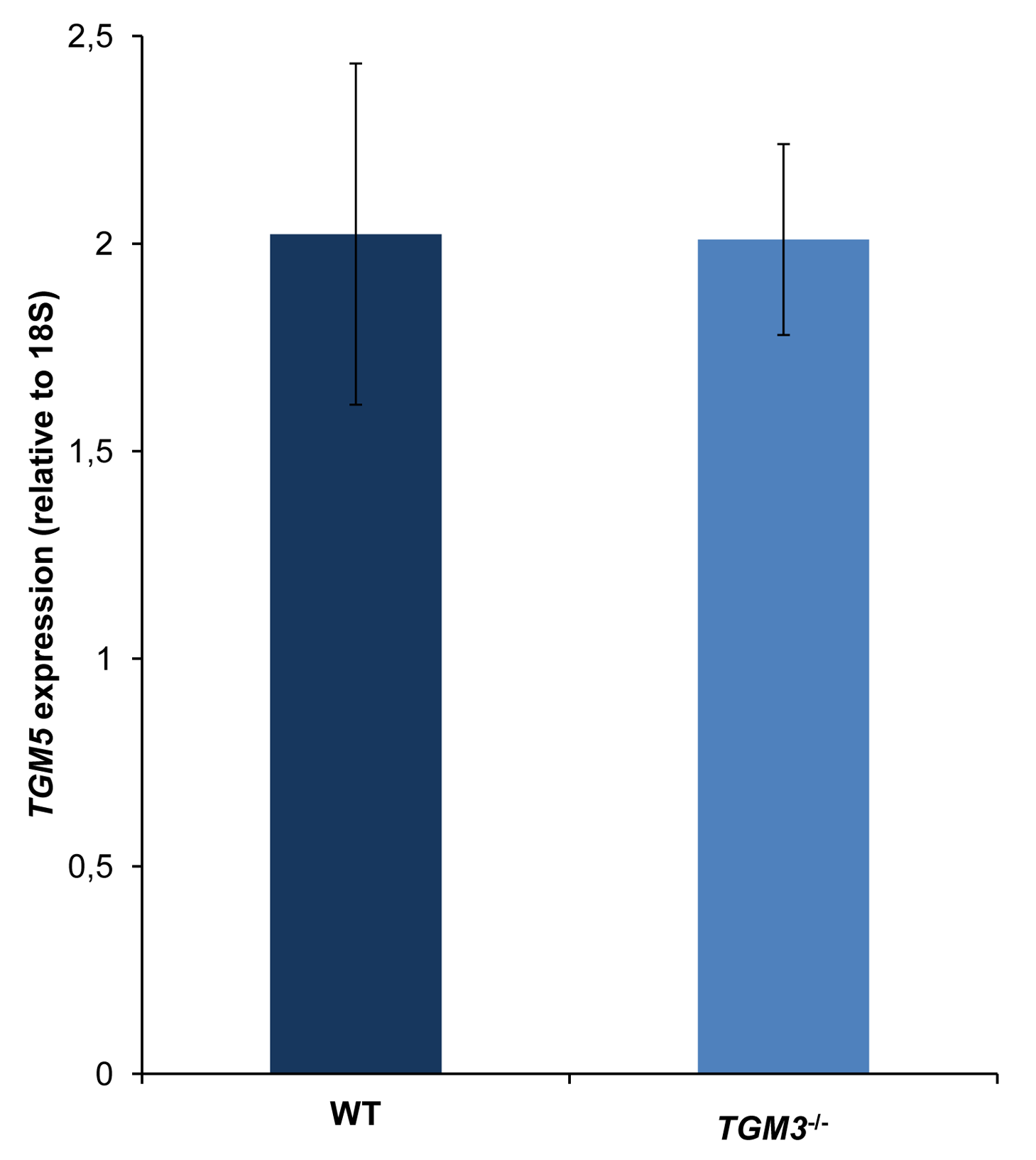

Supplement: Figure S3 — Analysis of TGase 5 RNA expression upon insertion. To check for any compensatory up-regulation in TGM5 message, total RNA from skin was reverse transcribed by random priming and analyzed by qPCR. Relative expression values (2ΔΔC(t)) for TGM5 were obtained by comparing it with the 18S amplification. No difference was observed between wild type (2.023, sd +/−0.411, n = 4) and TGM3−/− animals (2.01, sd +/−0.23, n = 4). (TIF) [file pone.0034252.s003.tif]
